# NCI677397 targeting USP24‐mediated induction of lipid peroxidation induces ferroptosis in drug‐resistant cancer cells

**DOI:** 10.1002/1878-0261.13574

**Published:** 2023-12-30

**Authors:** Shao‐An Wang, Yu‐Chih Wu, Feng‐Ming Yang, Feng‐Lin Hsu, Kuan Zhang, Jan‐Jong Hung

**Affiliations:** ^1^ School of Respiratory Therapy, College of Medicine Taipei Medical University Taiwan; ^2^ Cardiovascular Research Institute University of California, San Francisco CA USA; ^3^ Department of Biotechnology and Bioindustry Sciences National Cheng Kung University Tainan Taiwan

**Keywords:** cancer, ferroptosis, lipid peroxidation, small molecular inhibitor, USP24

## Abstract

Cancer represents a profound challenge to healthcare systems and individuals worldwide. The development of multiple drug resistance is a major problem in cancer therapy and can result in progression of the disease. In our previous studies, we developed small‐molecule inhibitors targeting ubiquitin‐specific peptidase 24 (USP24) to combat drug‐resistant lung cancer. Recently, we found that the USP24 inhibitor NCI677397 induced ferroptosis, a type of programmed cell death, in drug‐resistant cancer cells by increasing lipid reactive oxygen species (ROS) levels. In the present study, we investigated the molecular mechanisms and found that the targeting of USP24 by NCI677397 increased gene expression of most lipogenesis‐related genes, such as acyl‐CoA synthetase long‐chain family member 4 (*ACSL4*), and activated autophagy. In addition, the activity of several antioxidant enzymes, such as glutathione peroxidase 4 (GPX4) and dihydrofolate reductase (DHFR), was inhibited by NCI677397 treatment via an increase in protein degradation, thereby inducing lipid ROS production and lipid peroxidation. In summary, we demonstrated that NCI677397 induced a marked increase in lipid ROS levels, subsequently causing lipid peroxidation and leading to the ferroptotic death of drug‐resistant cancer cells. Our study provides new insights into the clinical use of USP24 inhibitors as ferroptosis inducers (FINs) to block drug resistance during chemotherapy.

## Graphical abstract

Wang *et al.* found that a novel USP24 inhibitor, NCI677397, as a ferroptosis inducer (FIN) promoted ferroptotic cell death in drug‐resistant cancers by increasing lipid peroxidation. Moreover, incomplete autophagy, HO‐1 induction and reduced anti‐oxidant levels by NCI677397 led to lipid ROS accumulation.

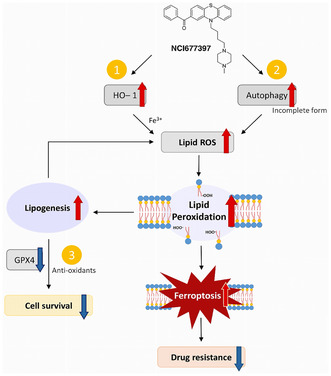

**Abbreviations**
A549‐T24: Taxol‐resistant A549 cells
ACSL4: acyl‐CoA synthetase long‐chain family member 4
ATCC: American Type Culture Collection
CE: cholesterol esters
DHFR: dihydrofolate reductase
FIN: ferroptosis inducer
GBM: glioblastoma multiforme
GPX4: glutathione peroxidase 4
HO‐1: heme oxygenase‐1
HSC: hepatic stellate cell
iTRAQ: isobaric tags for relative and absolute quantitation
OxPE: oxidation of phosphatidylethanolamine
PUFA‐PL: polyunsaturated fatty acid‐containing phospholipid
ROS: reactive oxygen species
TAM: tumor‐associated microenvironment
TMZ: temozolomide
UPS: ubiquitin‐proteasome system
USP24: ubiquitin‐specific peptidase 24

## 1 Introduction

Ubiquitin‐specific peptidase 24 (USP24), discovered in the context of genetic variation and associated with Parkinson's disease risk, is one of the largest deubiquitinating enzymes in the ubiquitin‐specific protease family and that regulates the ubiquitin modification of the substrates [1, 2, 3, 4]. By removing the ubiquitin moieties from a protein, USP24 increases protein stability to regulate the localization and interactions of its substrates, such as p53 [5], Mcl‐1 [6], p300 [7], DDB2 [8], Bax [9], and NCOA4 [10]. Thus, USP24 is involved in DNA damage repair, iron metabolism, cell death control, tumorigenesis, and transcription regulation. It has been reported that USP24 bound to Gasdermin B (GSDMB) via the activation of the STAT3 pathway promotes bladder cancer cell proliferation [11]. Moreover, USP24 inhibitors block B‐cell malignancies and the progression of T‐cell acute lymphoblastic leukemia [6]. Our previous studies have shown that USP24 is overexpressed in lung cancer and in the tumor‐associated microenvironment (TAM) and is involved in tumor malignancy and drug resistance [7, 12, 13]. Recently, we developed a novel USP24 small‐molecule inhibitor, NCI677397, to overcome Taxol‐induced drug‐resistant lung cancer [14]. However, the details of the molecular mechanisms underlying NCI677397 action in drug‐resistant cancers need to be clarified.

Autophagy is a regulated bulk degradation process in which cellular components, damaged proteins, and defective organelles are sequestered into double‐membrane transiently formed compartments that mature into autophagosomes and fuse with lysosomes, which ultimately leads to autophagosome cargo degradation [15]. The products broken down via autophagy are released into the cytosol for providing energy and/or nutrients for homeostasis and cellular remodeling during developmental programming [16]. Although basal autophagy is generally perceived to be hyperactivated, cytoprotective, or impaired autophagy contributes to various forms of regulated cell death, ferroptosis included [17, 18]. A regulated cell death modality, ferroptosis is associated with unrestricted lipid peroxidation and disrupted iron homeostasis [19]. Ferroptosis is a highly regulated reactive oxygen species (ROS)‐dependent type of cell death induced by lipid peroxidation, ROS generation, free iron overload and ultimately membrane damage [20]. Lipid peroxidation and iron overload are two biochemical hallmarks of ferroptosis and can result in pathologies. Acyl‐CoA synthetase long‐chain family member 4 (ACSL4) and lipoxygenases are two core enzymes that promote lipid peroxidation and then ferroptotic cell death [21, 22]. ACSL4 is vital for the generation of polyunsaturated fatty acid‐containing phospholipids (PUFA‐PLs), whereas lipoxygenases catalyze the oxidation of PUFA‐PLs, resulting in the production of lipid hydroperoxides and reactive aldehydes such as malondialdehyde (MDA) and 4‐hydroxynonenal (4‐HNE) [23]. Similarly, cytochrome P450 oxidoreductase (POR) is involved in lipid peroxidation related to ferroptosis [24]. Ferroptosis is controlled by three antioxidant axes, i.e., the FSP1/DHODH/CoQ10 axis, the GCH1/BH4/DHFR axis and the cysteine/GSH/GPX4 axis [25]. To date, a number of selective forms of autophagy, including clockophagy, mitophagy, ferritinophagy, lipophagy, and chaperone‐mediated autophagy, have been shown to participate in the induction of ferroptotic cell death by degrading organelles [26]. However, the relationship between NCI677397 and ferroptosis remains unclear.

In this study, we investigated the detailed mechanisms of NCI677397 in drug‐resistant cancers. We unexpectedly found that NCI677397 might be a novel ferroptosis inducer (FIN) because it destabilizes antioxidants and ABC transporters, leading to redox imbalance and cholesterol trafficking disruption, respectively, and ultimately resulting in the programmed cell death known as ferroptosis.

## 2 Materials and methods

### 2.1 Cell culture and transfection

Human lung adenocarcinoma epithelial cell line A549 (RRID:CVCL_0023) was obtained from the American Type Culture Collection (ATCC) were cultured with RPMI 1640 medium (Life Technologies, Carlsbad, CA, USA). Human primary GBM cell line Pt’3 was cultured in Dulbecco's modified Eagle's medium (DMEM) (Thermo Fisher Scientific, Waltham, MA, USA). Human U87MG (RRID:CVCL_0022, ATCC) and A172 (RRID:CVCL_0131, ATCC) GBM cells, were cultured in DMEM. DMEM and RPMI medium contain 10% fetal bovine serum, 100 μg·mL^−1^ streptomycin sulfate, and 100 U·mL^−1^ penicillin G sodium. All cell lines were maintained at 37 °C and 5% CO_2_. Taxol‐resistant A549 and TMZ‐resistant GBM cells were maintained in the same culture medium containing Taxol and TMZ (Sigma‐Aldrich, St. Louis, MO, USA), respectively. All experiments were performed with mycoplasma‐free cells. All cell lines were regularly checked for mycoplasma contamination and authenticated using short tandem repeat (STR) profiling to ensure the integrity and reliability of the cell lines. For transfection, plasmid (mRFP‐EGFP‐LC3) transfected with Polyjet (SignaGen) was used according to manufacturer's instructions for overexpression. LC3B and ACSL4 siRNAs were transfected into cells with DharmaFECT 1 Reagent (Dharmacon, Lafayette, CO, USA) for knockdown.

### 2.2 Human specimens and primary glioblastoma cells Pt#3

The use of human specimens was approved by the Institute Review Board (IRB)/Ethics Committee from the office of human research in Taipei Medical University (Taipei, Taiwan). The consent of each patient was obtained and approved by Taipei Medical University IRB protocols, No. 201402018. Pt#3 glioblastoma cells were isolated from the glioblastoma tissue of a male patient, who was treated and cared in the Taipei Medical University Hospital during August 2014 to July 2017. The study methodologies conformed to the standards set by the Declaration of Helsinki and the experiments were undertaken with the understanding and written consent of each subject.

### 2.3 Cell viability

Cells (2 × 10^4^ cells per well in a 24‐well plate) were treated with different doses of drugs for 4 days. For MTT assay, after the treatment, 300 μL of fresh medium containing 0.5 mg·mL^−1^ MTT reagent (Sigma‐Aldrich) was added to each well and incubated for 30 min at 37 °C. Medium was removed and the crystals were dissolved in 300 μL of DMSO (Sigma‐Aldrich). The absorbance was measured at 570 nm by using an iMark Microplate Absorbance Reader (Bio‐Rad, Hercules, CA, USA) [27]. For CCK‐8, 10 μL of CCK‐8 (Cell Counting Kit‐8, Dojindo, Kumamoto, Japan, CK04‐13, MD, USA) was added to each well and incubated for 1 h at 37 °C. The absorbance was measured at 450 nm.

### 2.4 Xenograft model

For the subcutaneous tumor model, TMZ‐resistant U87MG‐R cells (1 × 10^6^ cells in 50 μL of DMEM) were implanted into both dorsal flanks of male nonobese diabetic/severe combined immunodeficiency (NOD.CB17‐Prkdcscid/NcrCrl, NOD/SCID) mice (8 weeks old, BioLASCO Co., Ltd., Taipei, Taiwan). A month after transplantation, mice were administrated with TMZ (20 mg·kg^−1^) and NCI677397 (20 mg·kg^−1^) twice a week by intraperitoneal injection for another 8 weeks. After the treatment, the mice were then sacrificed and the tumors were excised by surgery to measure the weight and size. Tumor size was calculated according to the formula: 1/2 × long side × (short side)^2^ [27]. All mice housed five per cage in an air‐conditioned vivarium with free access to food and water. Throughout the study, a 12‐h light/dark cycle was maintained with lights on at 8 AM. All protocols for animal experiments were approved by the Institutional Animal Care and Use Committee of the Taipei Medical University (Taipei, Taiwan). Animal experiments were conducted under the IACUC number (LAC‐2020‐0371).

### 2.5 Western blotting

Cells were collected by sampling buffer and analyzed by electrophoresis. Proteins were transferred to polyvinylidene difluoride (PVDF, Millipore, Darmstadt, Germany) membrane and TBST buffer (10 mm Tris–HCl, pH 8.0, 150 mm NaCl and 0.05% Tween 20) containing 5% nonfat milk was used for blocking. Anti‐USP24 (Proteintech, Rosemont, IL, USA, 1 : 2000), anti‐LC3B (Cell Signaling Technology, Beverly, MA, USA, 1 : 2000), anti‐actin (Sigma‐Aldrich, 1 : 10 000), anti‐Bax (Proteintech, 1 : 2000), anti‐p53 (Millipore, Bradford, USA, 1 : 2000), anti‐ABCG1 (Genetex, Irvine, CA, USA, 1 : 1000), anti‐ABCG5 (Genetex, 1 : 1000), anti‐ABCG8 (Genetex, 1 : 1000), anti‐caspase 8 (Cell Signaling Technology, 1 : 1000), anti‐HMGCS1 (Cell Signaling Technology, 1 : 2000), anti‐FDFT1 (Genetex, 1 : 2000), anti‐FDPS (Genetex, 1 : 2000), anti‐FASN (Genetex, 1 : 1000), anti‐XBP1 (Genetex, 1 : 1000), anti‐ CHOP (Cell Signaling Technology, 1 : 1000), anti‐phospho‐eIF2α (Cell Signaling Technology, 1 : 1000), anti‐phospho‐IRE1α (Novusbio, Centennial, CO, USA, 1 : 1000), anti‐BiP (BD Biosciences, La Jolla, CA, USA, 1 : 1000), anti‐p62 (Genetex, 1 : 2000), anti‐HO‐1 (Genetex, 1 : 2000), anti‐ACSL3 (Genetex, 1 : 1000), anti‐ACSL4 (Genetex, 1 : 2000), anti‐GPX4 (Genetex, 1 : 1000), anti‐DHFR (Genetex, 1 : 1000) and anti‐SLC47A1 (Proteintech, 1 : 1000) were used for probing interested proteins. After incubated with primary antibodies, PVDF membranes were then incubated with secondary immunoglobulin antibodies linked with horse radish peroxidase (Millipore, 1 : 3000). ECL western blotting detection system (Millipore) and ChemiDoc‐it imager (UVP) were used for detecting signals.

### 2.6 Immunofluorescence

Cells were seeded in 6‐well plates with cover slips inside for 24 h, followed by NCI677397 treatment for another 24 h. Cover slips were removed and cells were fixed with 4% paraformaldehyde in 4 °C for 15 min. After fixation, cover slips were washed with PBS, and incubated with 0.2% Triton X‐100 in PBS for 5 min at room temperature. Cover slips then blocked with 1% bovine serum albumin (BSA) for 1 h, and stain with anti‐LC3B antibody (Cell Signaling Technology, 1 : 200) for 16 h at 4 °C. After washing with PBS, cells were stained with Alexa Fluor® 488 (Thermo Fisher Scientific, 1 : 200) for 1 h at room temperature, and mounted with 90% glycerol containing DAPI. Fluorescent images were photographed with a fluorescence microscope (Leica, Wetzlar, Germany). For Filipin III staining, cover slips stain with Filipin III (Sigma‐Aldrich) and PI (Thermo Fisher Scientific) for 2 h. For lipid peroxidation, C11‐BODIPY^581/591^ (Thermo Fisher Scientific), a commercial lipid peroxidation sensor, was used according to the manufacturer instructions. After drug treatment, cells in the dishes were stained by adding BODIPY reagent and DAPI staining solution into media. After 30 min, cells were monitored under fluorescence microscope (Leica) or flowcytometry. For intracellular iron assays, the intracellular iron level was detected by FerroOrange (Donjindo, Japan) using a ImageXpress Pico microscope. Cells were plated into dishes and incubated under the culture conditions pertinent to the experiment. At the time‐point of interest, the cells were treated with 1 μm FerroOrange staining for 30 min at 37 °C in the dark after washing with PBS 3 times. Finally, the cells were observed under a microscope [28].

### 2.7 RNA‐seq

For RNA quantification and qualification, RNA Purity and quantification were checked using SimpliNano™ – Biochrom Spectrophotometers (Biochrom, Holliston, MA, USA). For transcriptome sequencing, a total amount of 1 μL total RNA per sample was used as input material for the RNA sample preparations. Sequencing libraries were generated using KAPA mRNA HyperPrep Kit (KAPA Biosystems, Roche, Basel, Switzerland) following manufacturer's recommendations and index codes were added to attribute sequences to each sample. Briefly, mRNA was purified from total RNA using magnetic oligo‐dT beads. Captured mRNA was fragmented by incubating at a high temperature in the presence of magnesium in KAPA Fragment, Prime and Elute Buffer (1×). First strand cDNA was synthesized using random hexamer priming. Combined 2^nd^ strand synthesis and A‐tailing, which converts the cDNA:RNA hybrid to double‐stranded cDNA (dscDNA), incorporated dUTP into the second cDNA strand, and added dAMP to the 3′ ends of the resulting dscDNA. dsDNA adapter with 3′dTMP overhangs were ligated to library insert fragments to generate the library fragments carrying the adapters. In order to select cDNA fragments of preferentially 300~400 bp in length, the library fragments were purified with KAPA Pure Beads system (KAPA Biosystems, Roche). The library carrying appropriate adapter sequences at both ends was amplified using KAPA HiFi HotStart ReadyMix (KAPA Biosystems, Roche) along with library amplification primers. The strand marked with dUTP in not amplified, allowing strand‐specific sequencing. At last, PCR products were purified using KAPA Pure Beads system and the library quality was assessed on the Qsep 100 DNA/RNA Analyzer (BiOptic Inc., Taiwan) [29].

### 2.8 Quantitative proteomics—iTRAQ labeling

The proteomics experiments were assisted by Biotools Co., Ltd. (New Taipei City, Taiwan). For protein extraction, the cell pellets were lysed with lysis buffer (20 mm HEPES buffer, 0.1% SDS, 1 mm EDTA) and phenylmethylsulfonyl fluoride (PMSF) on ice. The cell lysates were further sonicated in Sonicator 3000 (Misonix, Farmingdale, NY, USA) (1 min; output level of 1; 3 s ON and 10s OFF) and centrifuged at 14 000 ***g*** at 4 °C for 15 min. The supernatants were transferred to new tube and protein concentration would be determined with BCA assay (Pierce, Thermo). For in‐sol digestion, the protein solutions were first diluted in 200 mm triethylammonium bicarbonate (TEABC) and then reduced with 5 mm tris‐(2‐carboxyethyl) phosphine (TCEP, Sigma Aldrich) at 60 °C for 45 min, followed by cysteine blocking with 10 mm methyl methanethiosulfonate (MMTS, Sigma Aldrich) at 25 °C for 30 min. Samples were digested with sequencing grade modified porcine trypsin (Promega, Madison, WI, USA) at 37 °C for 16 h. The peptides were labeled with iTRAQ reagent for 1 h at room temperature, pooled and dried by vacuum centrifugation. Sample was store at −20 °C until use. For LC–MS/MS analysis, the dried peptide mixtures were reconstituted in HPLC buffer A (0.1% formic acid) and loaded onto a reverse phase column (Zorbax 300SB C18, 0.3 × 5 mm; Agilent Technologies, Wilmington, DE, USA). The desalted peptides were then separated on a homemade column (Waters BEH 1.7 μm, 100 μm I.D. × 10 cm with a 15 μm tip) using a multistep gradient of HPLC buffer B (99.9% acetonitrile/0.1% formic acid) for 120 min with a flow rate of 0.3 μL·min^−1^. The LC apparatus was coupled with a 2D linear ion trap mass spectrometer (Orbitrap Elite; Thermo Fisher Scientific) operated using xcalibur 2.2 software (Thermo Fisher, San Jose, CA, USA). The full scan MS was performed in the Orbitrap over a range of 400–2000 Da and a resolution of 120 000 at *m*/*z* 400. The 16 data‐dependent MS/MS scan events (8 CID, 8 HCD) were followed by one MS scan for the 8 most abundant precursor ions in the preview MS scan. The *m*/*z* values selected for MS/MS were dynamically excluded for 80 s with a relative mass window of 15 ppm. The electrospray voltage was set to 2.0 kV, and the temperature of the capillary was set to 200 °C. MS and MS/MS automatic gain control were set to 1000 ms (full scan) and 300 ms (MS/MS), or 3 × 10^6^ ions (full scan) and 3 × 10^4^ ions (MS/MS) for maximum accumulated time or ions, respectively [29, 30].

### 2.9 Real‐time PCR

The RNA sample was extracted by TRIzol (Thermo Fisher Scientific), and 1 μg of total RNA was subjected to real‐time PCR reagent using Prime Script™ RT Reagent kit (Takara Bio. Inc, Shiga, Japan). The expression of each mRNA was determined using 2 × SYBR real‐time master mix (AB Sciex) and the specific primers. GAPDH was used as the internal control. SYBR green fluorescence was then monitored using an ABI 7000 Sequence Detection System (AB Sciex) [27]. Primer sequences are listed in Table S1.

### 2.10 Untargeted lipid metabolomics

For metabolites extraction, 200 μL water was added to each sample, after 30s vortex, the samples were frozen and thawed with liquid nitrogen for 3 times. Then, the samples were sonicated for 10 min in ice‐water bath. Add 480 μL MTBE: MEOH = 5 : 1 to the EP tube. After 30 s vortex, then the samples were sonicated for 10 min in ice‐water bath. Then the samples were incubated at −40 °C for 1 h and centrifuged at 3000 rpm (RCF = 900(***g***), *R* = 8.6 cm) for 15 min at 4 °C. 300 μL of supernatant was transferred to a fresh tube and dried in a vacuum concentrator at 37 °C. Then, the dried samples were reconstituted in 200 μL of 50% methanol in dichloromethane by sonication on ice for 10 min. The constitution was then centrifuged at 13 000 rpm (RCF = 16 200(***g***), *R* = 8.6 cm) for 15 min at 4 °C, and 100 μL of supernatant was transferred to a fresh glass vial for LC/MS analysis. The quality control (QC) sample was prepared by mixing an equal aliquot 20 μL of the supernatants from all of the samples. For LC–MS/MS analysis, it was performed using an UHPLC system (Vanquish, Thermo Fisher Scientific) with a UPLC HSS T3 column (2.1 mm × 100 mm, 1.8 μm) coupled to Q Exactive HFX mass spectrometer (Orbitrap MS, Thermo). The mobile phase A consisted of 40% water, 60% acetonitrile, and 10 mmol·L^−1^ ammonium format. The mobile phase B consisted of 10% acetonitrile and 90% isopropanol, which was added with 50 mL 10 mmol·L^−1^ ammonium format for every 1000 mL mixed solvent. The analysis was carried with elution gradient as follows: 0~1.0 min, 40% B; 1.0~12.0 min, 40~100% B; 12.0~13.5 min, 100% B; 13.5~13.7 min, 100~40% B; 13.7~18.0 min, 40% B. The column temperature was 55 °C. The auto‐sampler temperature was 4 °C, and the injection volume was 2 μL (pos) or 2 μL (neg), respectively. The QE mass spectrometer was used for its ability to acquire MS/MS spectra on data‐dependent acquisition (DDA) mode in the control of the acquisition software (Xcalibur 4.0.27, Thermo). In this mode, the acquisition software continuously evaluates the full scan MS spectrum. The ESI source conditions were set as following: sheath gas flow rate as 30 Arb, Aux gas flow rate as 10 Arb, capillary temperature 350 °C, full MS resolution as 120 000, MS/MS resolution as 7500, collision energy as 10/30/60 in NCE mode, spray Voltage as 4 kV (positive) or −3.8 kV (negative), respectively [31, 32, 33].

### 2.11 Estimation of H_2_O_2_ level

Cells were seeded on a 96‐well plate at a density of 5000 cells per well. After treatment, H_2_O_2_ level was detected by using a ROS‐Glo™ H_2_O_2_ Assay kit (Promega) according to the manufacturer's instruction. Samples were incubated with H_2_O_2_ Substrate Solution for 6 h, followed by the addition of ROS‐Glo™ detection solution and incubation for 20 min. Finally, read relative luminescence units (RLU) were estimated by a GloMax® Discover Microplate Reader (GM3000, Promega) [34].

### 2.12 Mitochondrial superoxide analysis

Mitochondria‐derived superoxide was estimated using MitoSOX® Red mitochondrial superoxide indicator (M36008, Thermo Fisher Scientific), and the cells were counterstained with DAPI (Abcam, Cambridge, UK). Live cells were imaged and quantified using an ImageXpress Pico automated cell imaging system (Molecular Devices, San Jose, CA, USA) [34].

### 2.13 Statistics

The data obtained were represented as means ± SEM. Two‐tailed unpaired Student's *t*‐test or two‐way ANOVA (animal experiments) were used to analyze the differences between the control and experimental groups. *P < 0.05, **P < 0.01, and ***P < 0.001 were considered significant in all comparisons.

## 3 Results

### 3.1 The novel specific USP24 inhibitor, NCI677397, induces TMZ‐resistant GBM cell death

**Fig. 1:**
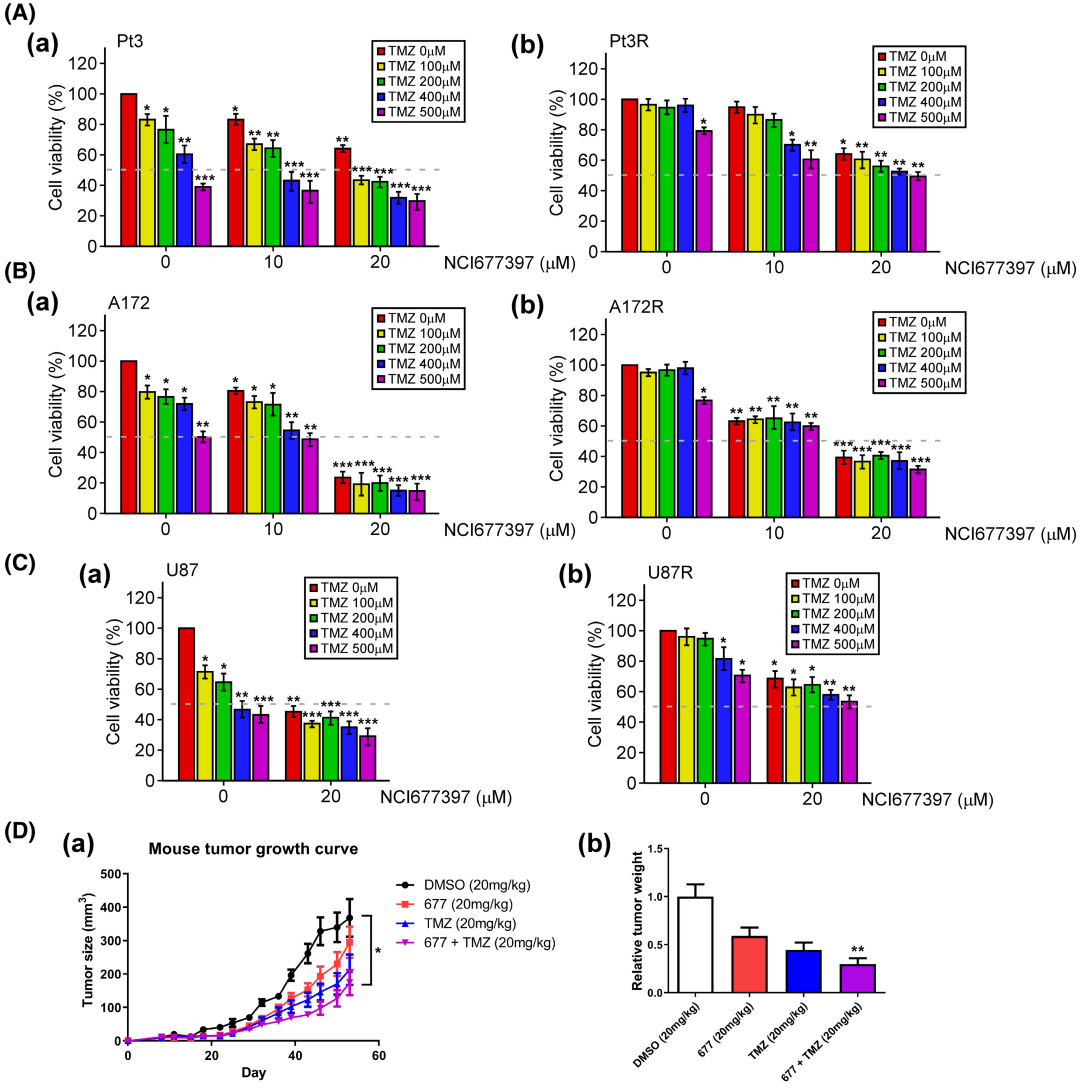
Suppressive effect of NCI677397 on TMZ‐sensitive or TMZ‐resistant glioblastomas *in vitro* and *in vivo*. (A–C) Viability of TMZ‐sensitive (Pt’3, A172 and U87) (A) and TMZ‐resistant (Pt’3R, A172R and U87R) (B) cells after NCI677397 or TMZ treatment was determined by MTT assay. Experiments were performed independently three times, the data are expressed as the mean ± SEM. and statistically analyzed by *t* test: *P < 0.05; **P < 0.01; ***P < 0.001. The dashed lines indicate a 50% reduction in the MTT assay. (D) Fourteen days after subcutaneous transplantation with U87R cells (1 × 10^6^), mice were administered 20 mg·kg^−1^ NCI677397 or TMZ via intraperitoneal injection for 7 weeks (2 times per week). The tumor size (A) and weight (B) were measured. The data are expressed as the mean ± SEM. and statistically analyzed by *t* test: *P < 0.05, **P < 0.01.

Resistance to therapeutic treatment is the main cause of the death of cancer patients, particularly glioblastoma (GBM) patients. The standard care protocol for glioblastoma is temozolomide (TMZ)‐mediated chemotherapy conjugated with radiotherapy [35]. Our previous studies indicated that USP24 promotes drug resistance during cancer therapy and leads to the development of a novel USP24‐specific small‐molecule inhibitor, NCI677397, to suppress drug resistance, especially in lung cancer [14]. The tumor‐suppressive effect of TMZ is restricted to a window of opportunity that is reduced by the high frequency of glioblastoma recurrence [36]. To understand whether the USP24 inhibitor can kill TMZ‐resistant GBM cells, we evaluated the effect of NCI677397 on the survival of TMZ‐sensitive and TMZ‐resistant glioblastoma cells (Fig. 1A–C). We compared the therapeutic effect of NCI677397 with TMZ. The results showed that TMZ treatment decreased the survival of TMZ‐sensitive GBM cells (Pt’3, A172 and U87 cells) but not TMZ‐resistant GBM cells (Pt’3R, A172R and U87R cells). However, NCI677397 (10 and 20 μm) combined with TMZ treatment significantly decreased the viability of TMZ‐sensitive GBMs and TMZ‐resistant GBM cells. To determine whether NCI677397 exhibits tumor‐suppressive effect *in vivo*, we generated a TMZ‐resistant cell (U87R) xenograft mouse model **(**Fig. 1D **)**. Fourteen days after we transplanted U87R cells into mice, we monitored tumor size. Notably, intraperitoneal administration of NCI677397 combined with TMZ markedly and significantly inhibited tumor growth, leading to smaller tumor size (Fig. 1D,a) and lower tumor weight (Fig. 1D,b) than those of tumors in mice receiving other treatment. Taken together, the results indicated that the USP24 inhibitor, NCI677397, worked synergistically with TMZ to induce resistant GBM cell death.

### 3.2 NCI677397 induces autophagy, not apoptosis, in both lung and brain cancers

**Fig. 2:**
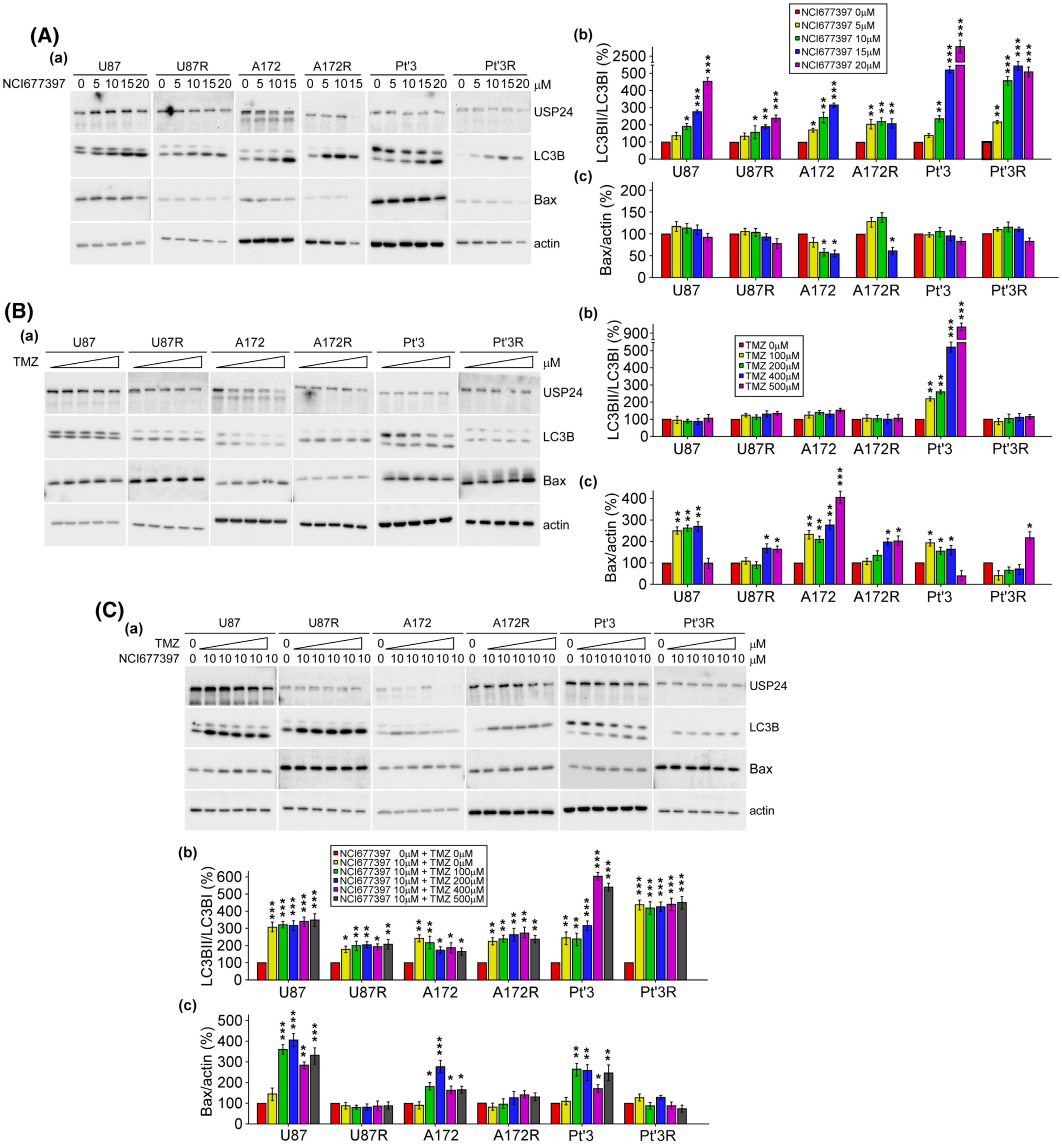
NCI677397 induces autophagy, not apoptosis, in brain and lung cancer cells. (A–C) The levels of LC3B and Bax in TMZ‐sensitive (U87, A172 and Pt’3) and TMZ‐resistant (U87R, A172R and Pt’3R) cells after NCI677397 (A) or TMZ (B) or in combination (C) were measured by western blotting with various antibodies. The LC3B (b) and Bax (c) levels were quantitated based on three independent experiments and statistically analyzed by *t* test: *P < 0.05, **P < 0.01, ***P < 0.001.

**Fig. 3:**
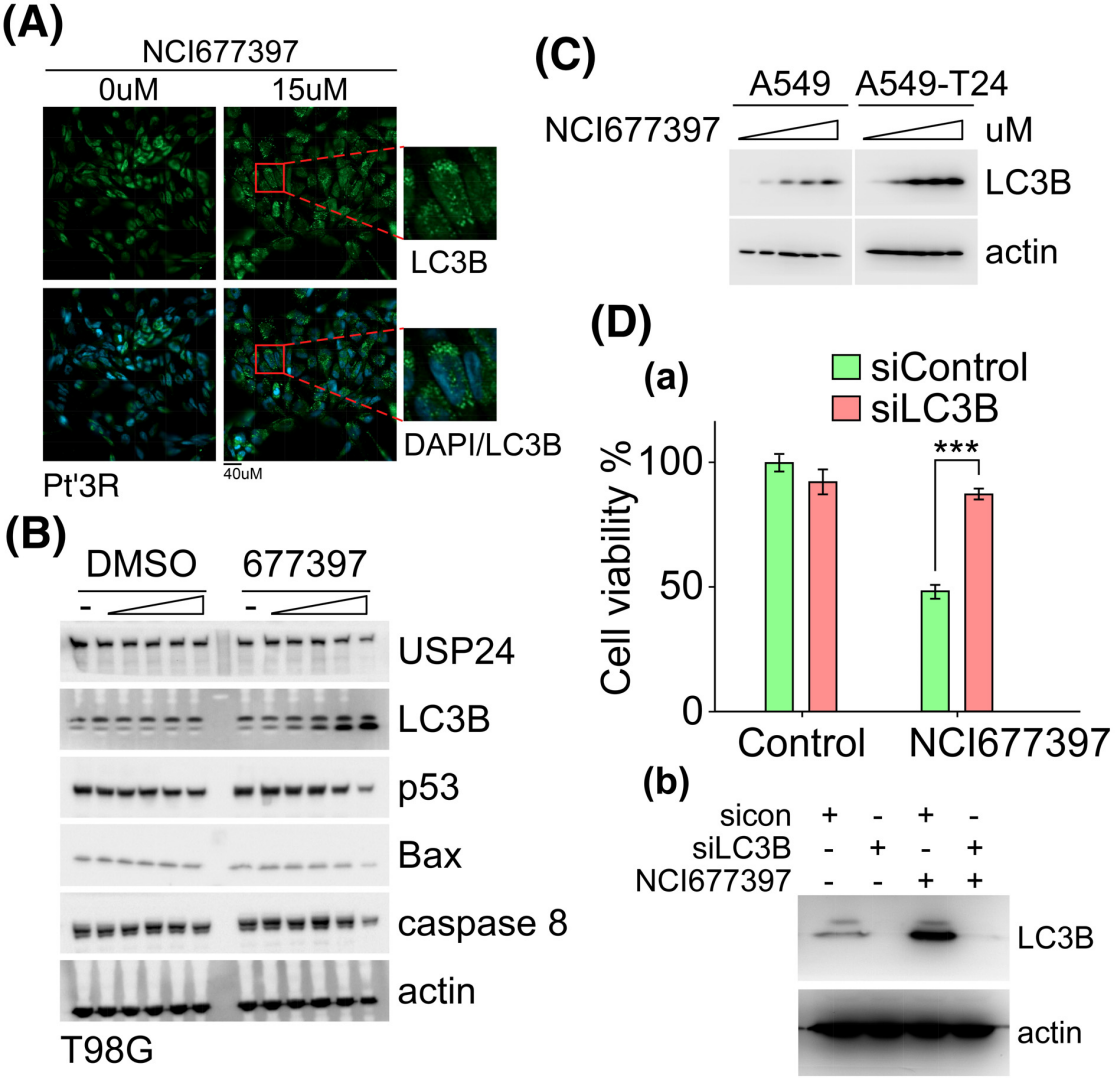
NCI677397 induced cell death via autophagy. (A) After 24 h in the presence or absence of NCI677397 for 24 h, Pt’3R cells were stained with an anti‐LC3B antibody for immunofluorescence. Scale bar, 40 μm. (B) The levels of LC3B, Bax, p53 and caspase 8 in T98G cells with or without NCI677397 treatment were measured by western blotting. (C) The level of LC3B in A549 or A549‐T24 cells with or without NCI677397 treatment was measured by western blotting. (D) After transfection LC3B siRNA and then treated with NCI677397, cell viability was studied by CCK‐8 assay (a) and cell lysates were analyzed by western blotting (b). Experiments were performed independently three times, the data are expressed as the mean ± SEM. and statistically analyzed by *t* test: ***P < 0.001.

Next, we investigated the molecular mechanisms by which NCI677397 leads to resistant brain cancer cell death and unexpectedly found that NCI677397 increased the levels of autophagosome‐associated lipidated form of LC3B (LC3B‐II), which indicated an increase in autophagy, but not an increase in the levels of Bax, an apoptotic marker, in both TMZ‐sensitive and TMZ‐resistant GBM cells (Fig. 2A). Moreover, we confirmed the effect of TMZ on autophagy and apoptosis, with the results indicating that TMZ could induced apoptosis but not autophagy, except in Pt’3 cells (Fig. 2B). In summary, a synergistic effect was observed when NCI677397 combined with TMZ, increasing autophagic and apoptotic cell death in TMZ‐sensitive and TMZ‐resistant GBMs cells (Fig. 2C). The ability of NCI677397 to regulate autophagy was confirmed by immunofluorescence assay with an LC3B antibody in Pt’3R cells (Fig. 3A). Specifically, treatment with the USP24 inhibitor led to the significant accumulation of LC3B puncta in autophagy machinery. Furthermore, NCI677397‐treated T98G cells, which are TMZ‐resistant GBM cells with high MGMT expression, also markedly increased autophagy, and a similar effect was observed in lung cancer cells, namely, A549 and Taxol‐resistant A549 (A549‐T24) cells (Fig. 3B,C). These data confirmed that the USP24 inhibitor is a positive regulator of autophagy in cancer cells. In addition, to determine whether NCI677397 induces autophagic cell death, cell viability was evaluated by CCK‐8 assay. As shown in Fig. 3D, NCI677397 markedly decreased cell viability, but this effect was reversed by knocking down LC3B, demonstrating that NCI677397 induced cell death via autophagy.

### 3.3 NCI677397 induces the biosynthesis of cholesterol and fatty acid in cancer cells

**Fig. 4:**
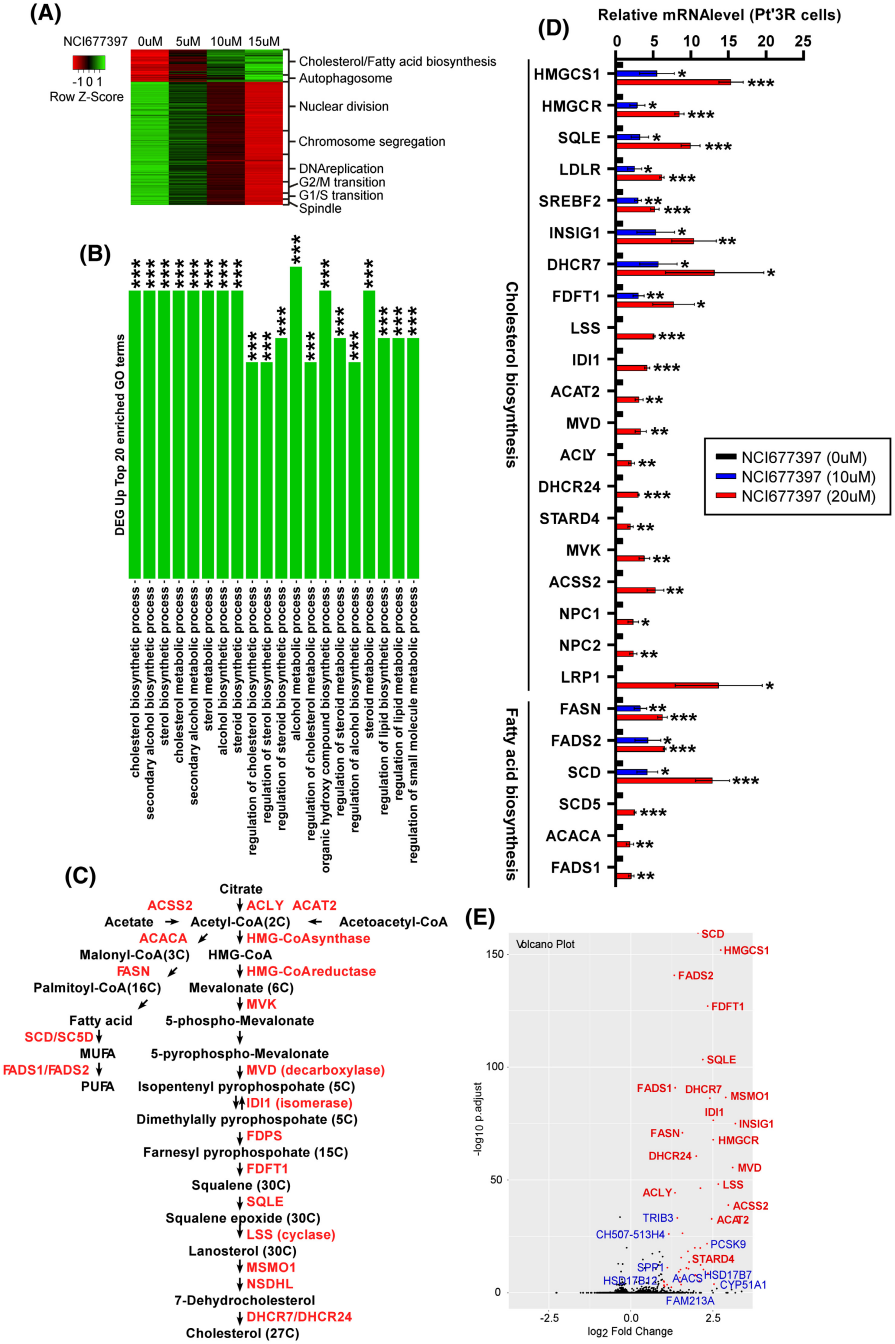
NCI677397 induces cholesterol and fatty acid biosynthesis by RNAseq analysis. (A) Heatmap showing the changes in the expression of mRNAs in NCI677397‐treated Pt’3R cells, including transcripts related to cholesterol, fatty acid biosynthesis, autophagosome, and cell cycle‐related genes in RNA‐seq analysis (*n* = 3). (B) The upregulation, as identified via an RNA‐seq analysis of NCI677397‐treated Pt’3R cells, of the 20 most enriched genes in a Gene Ontology (GO) analysis of biological process. ***adjusted *P*‐value <0.001. (C) The cholesterol and fatty acid biosynthesis pathways and related enzymes (red). (D) The qPCR measurements of cholesterol and fatty acid biosynthesis‐related genes in NCI677397‐treated Pt’3R cells based on three independent experiments, the data was expressed as the mean ± SEM. and a statistical analysis by *t* test: *P < 0.05, **P < 0.01, ***P < 0.001. (E) The Volcano plot summarizing the effect of NCI677397 treatment in A549‐T24 cells on the respective protein abundances as determined via RNA‐seq analysis (*n* = 3).

**Fig. 5:**
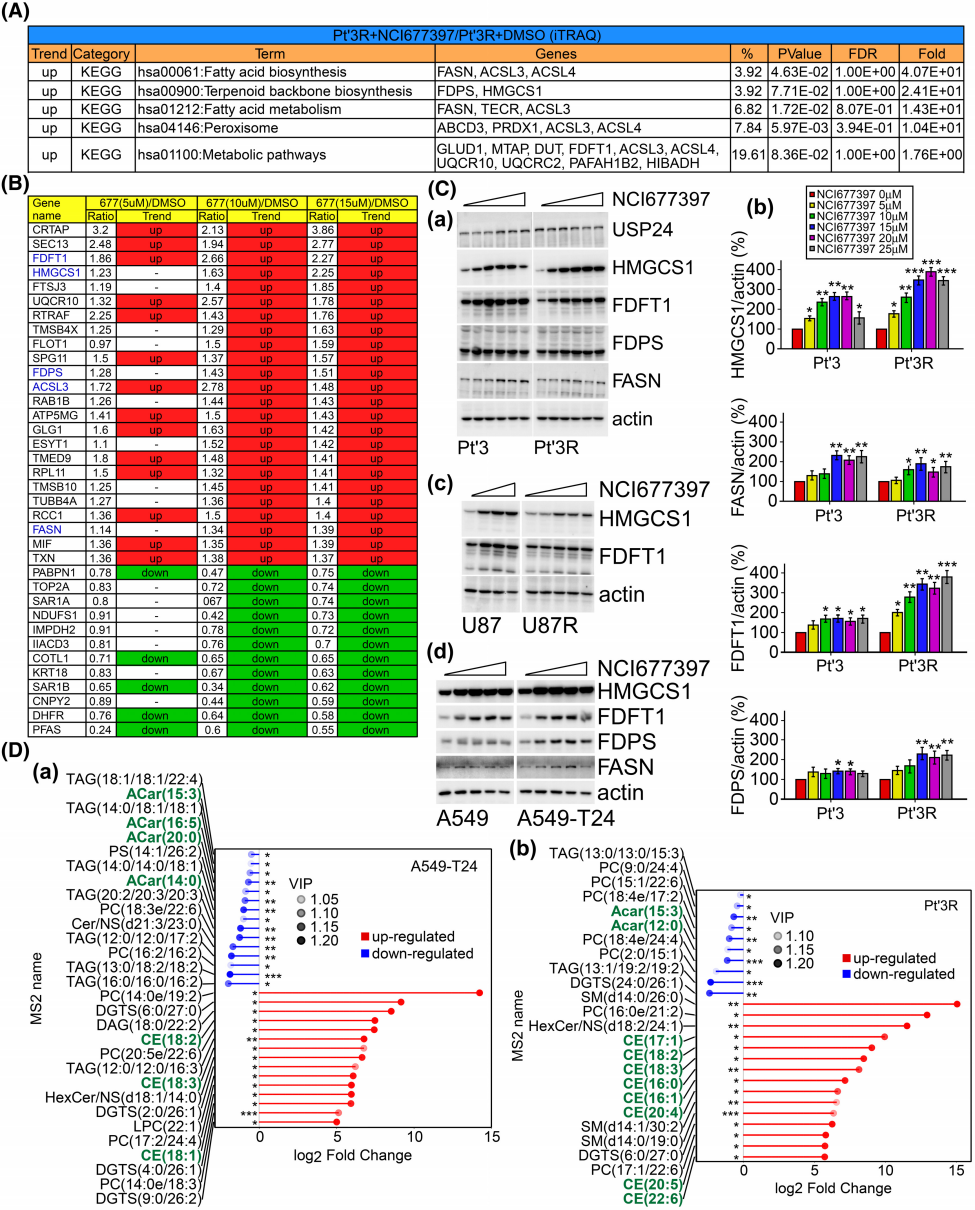
NCI677397 Induces cholesterol and fatty acid biosynthesis by MASS analysis. (A) Enriched Kyoto Encyclopedia of Genes and Genomes (KEGG) pathways of differentially expressed genes in iTRAQ analysis (*n* = 3) and statistically analyzed by *t* test. (B) Protein quantification by iTRAQ analysis. Proteins that were downregulated or upregulated by NCI677397 in a dose‐dependent manner (*n* = 3). Blue text (genes) indicates ferroptosis‐related genes. (C) After treatment with NCI677397 for 24 h, Pt’3, Pt’3R (a), U87, U87R (c), A549 and A549‐T24 (d) cells were harvested, and proteins from lysate cells were measured by western blotting with the indicated antibodies. Quantitative levels (normalized to the level of Actin) of the indicated proteins in Pt’3 or Pt’3R cells based on three independent experiments, the data was expressed as the mean ± SEM and statistically analyzed by *t* test: *P < 0.05, **P < 0.01, ***P < 0.001 (b). (D) Important metabolites indicative of the fate of NCI677397‐treated A549‐T24 (a) and GBM (b) cells as determined via UHPLC‐QTOF‐MS are displayed in a matchstick diagram, and statistically analyzed by *t* test: *P < 0.05; **P < 0.01; ***P < 0.001. The abscissa shows the log‐transformed change multiple, and the dot color intensity represents the VIP value size (*n* = 3).

We wondered whether NCI677397 induces autophagic cancer cell death and wanted to understand the detailed mechanisms underlying the effects of the USP24 inhibitor; therefore, we performed an RNA‐seq analysis to generate a global mRNA expression profile of NCI677397‐treated in Pt’3R cells (Fig. 4). The data were used to generate a heatmap, which showed that the gene expression levels related to cholesterol and fatty acid biosynthesis and autophagosomes were upregulated in NCI677397‐treated cells, while the expression levels of genes related to the cell cycle, cell division and DNA replication were downregulated (Fig. 4A). In addition, upregulation of differentially expressed genes (DEGs) after NCI677397 treatment were subjected to a Gene Ontology (GO) enrichment analysis, which revealed that the 20 most enriched pathway were almost all of them involved in the cholesterol biosynthetic process (Fig. 4B). Therefore, we further analyzed the upregulated DEGs that encoded enzymes involved in cholesterol and fatty acid biosynthesis and identified the cholesterol and fatty acid biosynthesis pathway (Fig. 4C). Interestingly, the red fonts refer to cholesterol and fatty acid biosynthesis enzymes, all of which were upregulated after NCI677397 treatment according to the RNA‐seq data (Fig. 4C). Moreover, we reconfirmed the RNA‐seq data by performing qPCR with samples prepared from Pt’3R cells after NCI677397 treatment, and the results were similar to those obtained by analyzing RNA‐seq data (Fig. 4D). Furthermore, a volcano plot of DEGs in Taxol‐resistant A549 lung cancer cells treated with the USP24 inhibitor showed that cholesterol and fatty acid biosynthesis enzymes were upregulated, and they are presented in red (Fig. 4E). In addition, to further investigate the mechanism underlying NCI677397‐induced cancer cell death, we analyzed alterations of the protein expression profile by using isobaric tags for relative and absolute quantitation (iTRAQ) analysis. A KEGG pathway analysis revealed that fatty acid and terpenoid backbone biosynthesis, which is mediated upstream of the cholesterol biosynthesis pathway, were upregulated in NCI677397‐treated Pt’3R cells (Fig. 5A). The protein expression levels which were significantly downregulated and upregulated in the Pt’3R cells treated with NCI677397 are listed in Fig. 5B. Similar to the RNA‐seq data analysis, iTRAQ showed that NCI677397 induced cholesterol and fatty acid biosynthesis enzymes, such as FDFT1, HMGCS1, FDPS, and FASN, presented in blue (Fig. 5B). We confirmed the results of iTRAQ by western blotting with protein samples from sensitive and resistant brain and lung cancer cells and demonstrated that NCI677397 markedly increased the expression of FDFT1, HMGCS1, FDPS, and FASN (Fig. 5C). Finally, to make clarify whether NCI677397 induces cholesterol biosynthesis, we executed an untargeted lipid metabolomic analysis with brain and lung cancer cells. The results indicated that NCI677397 upregulated cholesterol esters (CEs), for example, CE(18 : 2), CE(18 : 3), CE(18 : 1), and CE(20 : 4), which are cholesterol‐storage lipids in cells (Fig. 5D). Overall, our results illustrated that NCI677397 indeed enhanced cholesterol biosynthesis in cancer cells.

### 3.4 NCI677397 induces ferroptosis mediated via lipid ROS

**Fig. 6:**
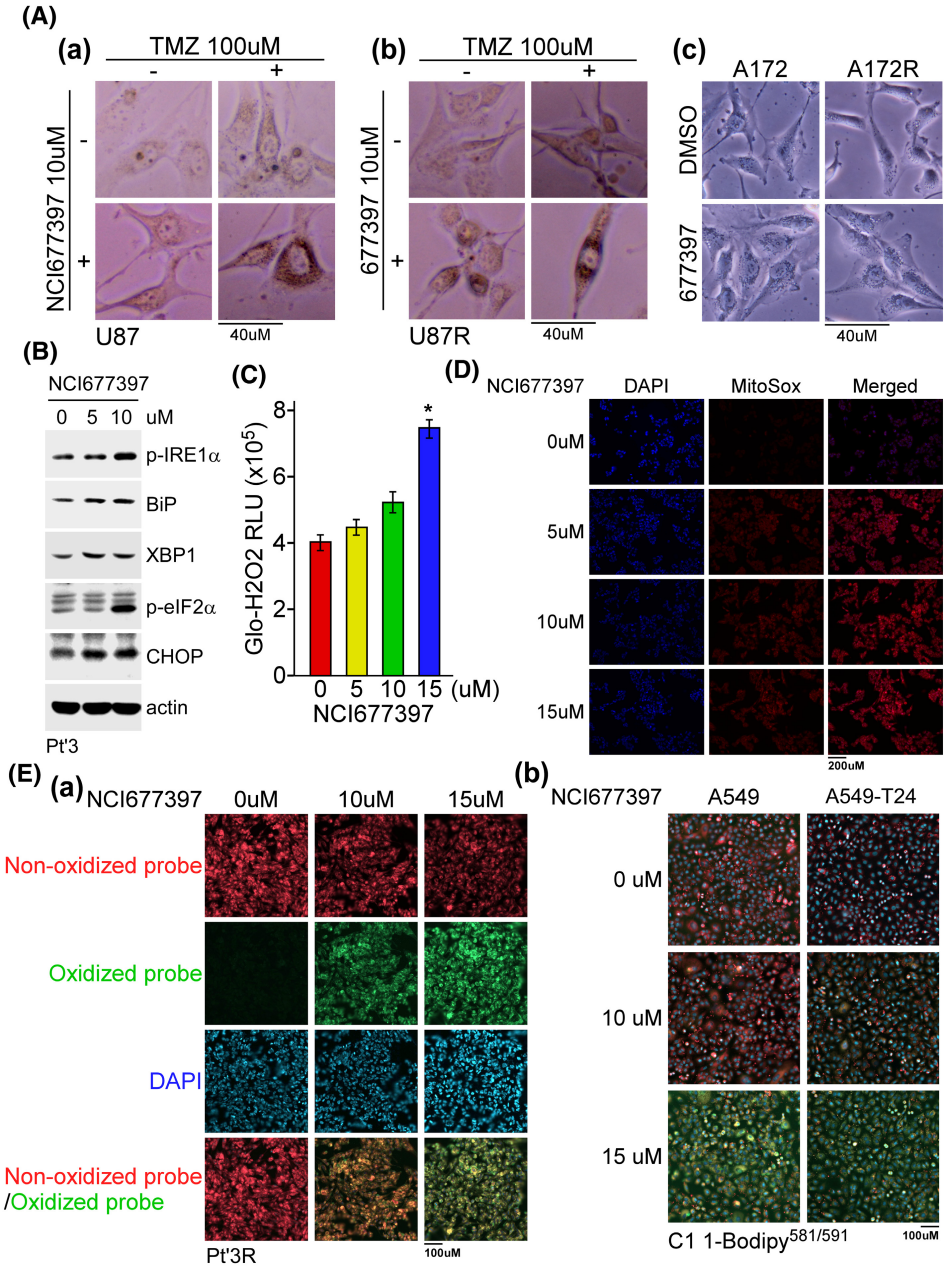
NCI677397 induces lipid ROS. (A) Representative cell images of U87, U87R, A172 and A172R cells treated with NCI677397 or TMZ (*n* = 2). Scale bar, 40 μm. (B) Cells collected 1 day after treatment with NCI677397 at different concentrations. The levels of proteins from cell lysates were measured by western blotting with the indicated antibodies (*n* = 2). (C) After treatment with NCI677397 for 24 h, the cells were harvested, and the relative H_2_O_2_ levels were estimated. After three independent experiments, the data was expressed as the mean ± SEM and the statistical analysis was performed by *t* test: *P < 0.05. (D) After treatment with NCI677397 for 24 h, live Pt’3R cells were stained with the Mito‐SOX mitochondrial free radical detector (*n* = 2). Scale bar, 200 μm. (E) After 24 h of treatment with NCI677397, lipid ROS was estimated by staining Pt’3R (a), A549 and A549‐T24 (b) cells with C11‐BODIPY^581/591^ reagent. The immunofluorescence signal representing peroxidase lipid (green) was photographed with a fluorescent microscope (*n* = 3). Scale bar, 100 μm.

**Fig. 7:**
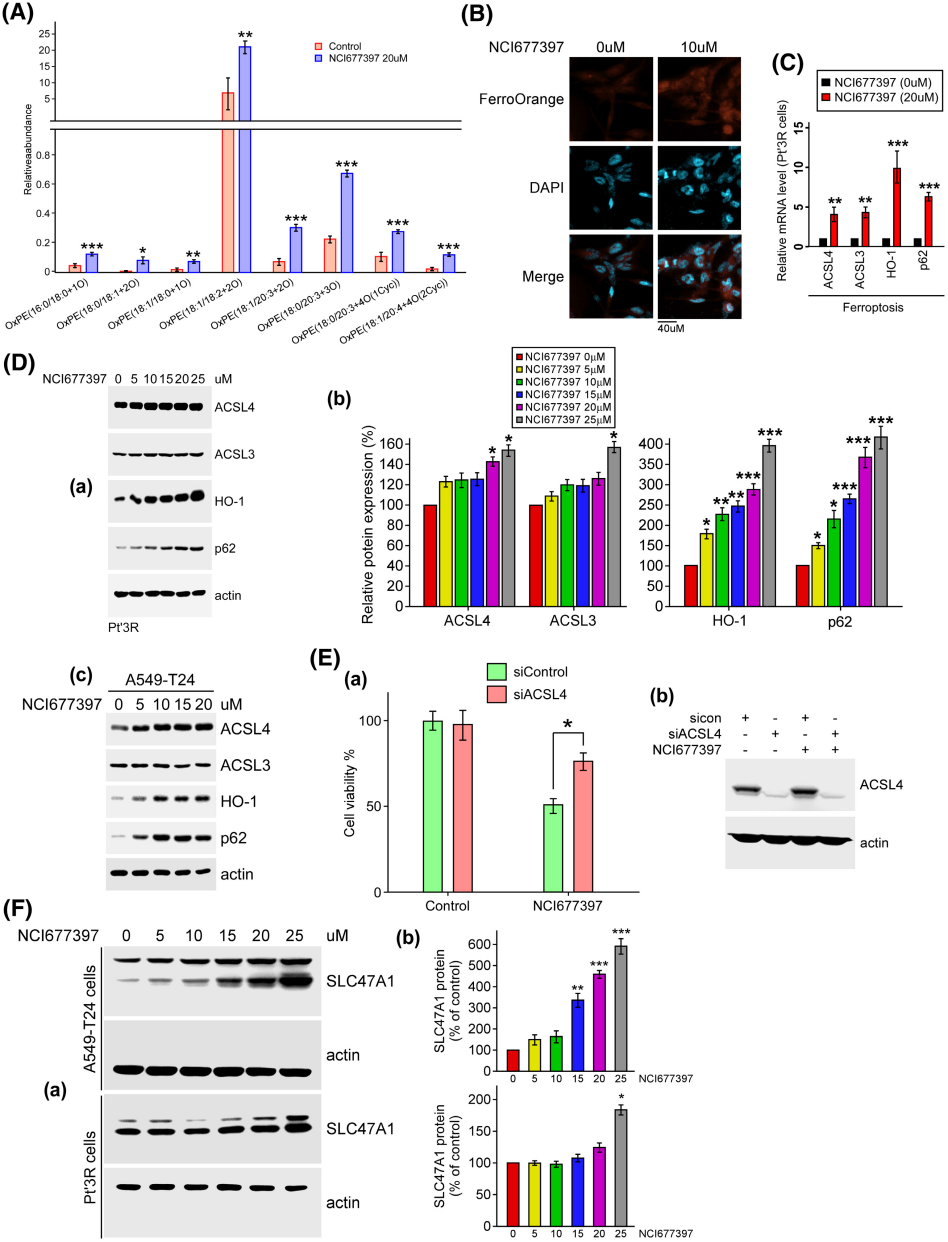
NCI677397 induces ferroptosis. (A) Quantification of oxidized phosphatidylethanolamine (OxPE) in NCI677397‐treated cells performed via untargeted lipidomics analysis independently three times, the data was expressed as the mean ± SEM (*P < 0.05; **P < 0.01; ***P < 0.001). (B) NCI677397‐treated cells stained with FerroOrange dye were used for intracellular iron measurement or DAPI (*n* = 3). Scale bar, 40 μm. (C) qPCR measurement of ferroptosis‐related gene levels in NCI677397‐treated Pt’3R cells. After three independent experiments, the data was expressed as the mean ± SEM (**P < 0.01; ***P < 0.001). (D) The levels of proteins from lysates of Pt’3R (A) or A549‐T24 (c) cells after treatment with different doses of NCI677397 were measured by western blotting with the indicated antibodies, and the proteins (B) from three independent experiments were quantitated, the data was expressed as the mean ± SEM and statistically analyzed by *t* test: *P < 0.05, **P < 0.01, ***P < 0.001. (E) After transfection with ACSL4 siRNA and then treatment with NCI677397, cell viability was studied by CCK‐8 assay (A) and cell lysates were analyzed by western blotting (B). Experiments were performed independently three times, and the data are expressed as the mean ± SEM (*P < 0.05). (F) The levels of proteins from lysates of A549‐T24 or Pt’3R cells after treatment with different doses of NCI677397 were measured by western blotting with the indicated antibodies (A), and the levels of proteins from three independent experiments were quantitated, the data was expressed as the mean ± SEM and statistically analyzed by *t* test: *P < 0.05, **P < 0.01, ***P < 0.001 (b).

To date, the reason that NCI677397 induces autophagy activation and cholesterol biosynthesis and leads to cancer cell death remains unclear. To further understand how NCI677397 kills the cancer cells, we evaluated whether NCI677397 enhances ER stress or affects the levels of reactive oxygen species (ROS) in cancer cells. We found that either TMZ or NCI677397 treatment, and especially, when they were administered in combination, induced particle accumulation near the nucleus in GBM cells, possibly at the ER or Golgi (Fig. 6A). Moreover, as shown in Fig. 6B, the levels of p‐IRE1α, Bip, XBP1, p‐eIF2α and CHOP, all of which are markers of ER stress, were increased by NCI677397 treatment in a dose‐dependent manner. For ROS measurements, we found that NCI677397 slightly increased the H_2_O_2_ level and mitochondrial ROS levels shown in Fig. 6C,D, respectively, but profoundly increased lipid ROS (oxidized lipid) levels in brain cancer and lung cancer cells (Fig. 6E), suggesting that NCI677397 promoted lipid peroxidation in cancer cells. Subsequently, we evaluated the results of a lipid metabolomic analysis and found that oxidation of phosphatidylethanolamine (OxPE) was induced by NCI677397 (Fig. 7A), implying that NCI677397 might promote ferroptosis, which is a type of programmed cell death dependent on iron and characterized by the accumulation of lipid peroxides [37]. Hence, we measured the intracellular iron levels on the basis of FerroOrange intensity in images and found that NCI677397 treatment led to a much higher level of Fe^2+^ than control (Fig. 7B). According to the iTRAQ data (Fig. 5A,B), in NCI677397‐treated Pt’3R cells, protein levels of ACSL3 and ACSL4 (acyl‐coenzyme A synthetase long‐chain family member), which are ferroptotic markers, were increased. In particular, ACSL4, which ligates free polyunsaturated fatty acids (PUFAs) with coenzyme A to produce PUFA‐CoAs, which are subsequently incorporated into phospholipids (PLs) and are required for ferroptotic cancer cell death [23]. In addition, we confirmed the increase in mRNA and protein levels of ACSL3 and ACSL4 in brain and lung cancer cells, which was similar with the iTRAQ data (Fig. 7C,D), illustrating that ferroptotic markers are upregulated by NCI677397. Next, we want to know whether NCI677397 induced ferroptotic cell death dependent on ACSL4 induction. The results showed that knockdown of ACSL4 reversed the anticancer effect of NCI677397 (Fig. 7E). Unexpectedly, we found that heme oxygenase‐1 (HO‐1), which consequently contributes to ferroptosis, and p62/SQSTM1, which is an autophagosome cargo protein, were also obviously increased by NCI677397 treatment (Fig. 7D). Furthermore, recent studies showed that SLC47A1 is a primary regulator of PUFA‐esterified cholesterol esters as a regulator of lipid remodeling during ferroptosis [38, 39]. Here, the data indicated that NCI677397 also induced SLC47A1 upregulation in both A549‐T24 and Pt3′R cells (Fig. 7F). Taken together, the data revealed that NCI677397 targeting USP24 function as a novel ferroptosis inducer (FIN), which was consistent with recent reports describing it as a novel treatment for reversing cancer cell drug resistance [40].

### 3.5 NCI677397 induces protein instability of ABC transporters and antioxidants

**Fig. 8:**
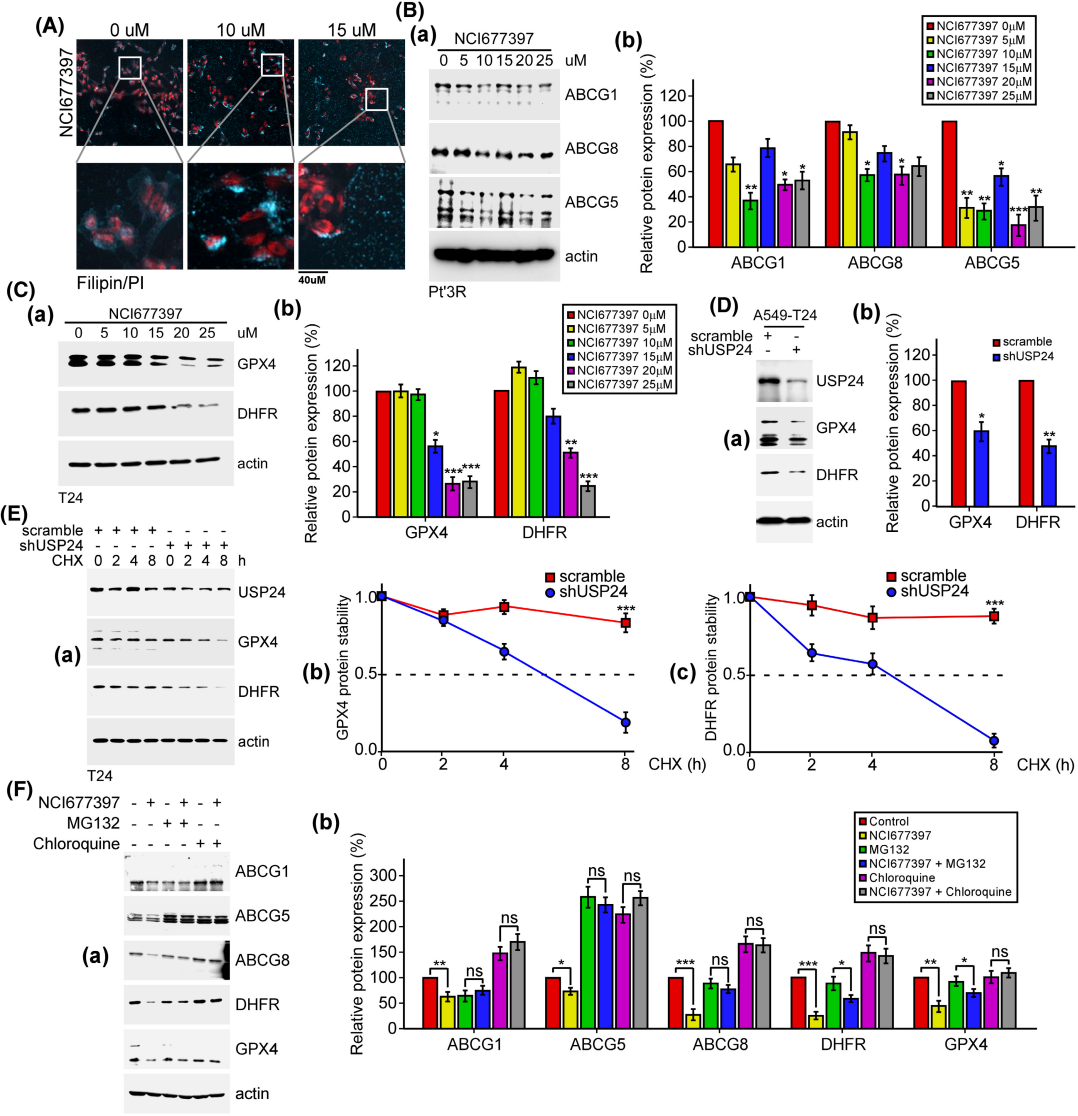
NCI677397 induces the protein instability of ABC‐transporters and antioxidants. (A) NCI677397‐treated cells stained with filipin for the detection of cholesterol or with PI for the detection of nuclei (*n* = 3). Scale bar, 40 μm. (B) The levels of proteins from the lysates of Pt’3R cells treated with different doses of NCI677397 were measured by western blotting with the indicated antibodies (A), and the indicated proteins (B) from three independent experiments were quantitated and statistically analyzed by *t* test: *P < 0.05, **P < 0.01, ***P < 0.001. (C) The levels of proteins from lysates of A549‐T24 cells treated with different doses of NCI677397 were measured by western blotting with the indicated antibodies (A), and the indicated proteins (B) from three independent experiments were quantitated, the data was expressed as the mean ± SEM and statistically analyzed by *t* test: *P < 0.05, **P < 0.01, ***P < 0.001. (D) A549‐T24 cells with USP24 knockdown were collected, and the levels of proteins were measured by western blotting with the indicated antibodies (A). The indicated proteins (B) from three independent experiments were quantitated, the data was expressed as the mean ± SEM and statistically analyzed by *t* test: *P < 0.05, **P < 0.01. (E) After knockdown of USP24, the proteins from A549‐T24 cells treated with cycloheximide for different durations were measured by western blotting with antibodies against GPX4 and DHFR (A), and the indicated proteins (b–c) from three independent experiments were measured, the data was expressed as the mean ± SEM and statistically analyzed by *t* test: ***P < 0.001. The dashed lines indicate a 50% reduction in the protein level. (F) The levels of proteins from lysates of A549‐T24 cells co‐treated with NCI677397 and MG132 or chloroquine were measured by western blotting with the indicated antibodies (a), and the indicated proteins (b) from three independent experiments were quantitated, the data was expressed as the mean ± SEM and statistically analyzed by *t* test: *P < 0.05, **P < 0.01, ***P < 0.001.

Next, we clarified the mechanisms by which NCI677397 induces cholesterol biosynthesis, autophagy activation, and ferroptosis. First, we analyzed the trafficking of cholesterol in Pt’3R cells using filipin fluorescent probes specifically binding cholesterol and revealed that cholesterol was homogenously distributed in the plasma membrane in normal cells, while cholesterol accumulated at the ER or Golgi in NCI677397‐treated cells (Fig. 8A), suggesting that NCI677397 obstructs cholesterol transportation from the ER/Golgi to the plasma membrane. Previous studies reported that the ABC transporter G family (ABCG1/5/8) functioned by facilitating cholesterol transport from the endoplasmic reticulum to the plasma membrane [41, 42, 43], and our recent study also revealed that USP24 stabilized ABCG2 to enhance the efflux of Taxol from cancer cells, resulting in drug resistance during cancer therapy [14]. Therefore, we expectedly found that NCI677397 inhibiting USP24 led to a decrease in the protein levels of ABCG1/5/8 (Fig. 8B). Ferroptosis is a novel form of regulated cell death and is characterized by iron dependence and disruption of the intracellular redox balance [44]. On the basis of the iTRAQ data, we found that the level of the important redox enzyme, DHFR, a recently discovered negative regulator of ferroptosis, was decreased by NCI677397 treatment in a dose‐dependent manner (Fig. 5B). Another essential redox enzyme involved in ferroptosis is GPX4, which is the main regulator of ferroptosis [45], and we found that NCI677397 reduced the protein level of GPX4 in addition to DHFR (Fig. 8C). Moreover, knocking down USP24 in A549‐T24 cells decreased the protein levels of both GPX4 and DHFR (Fig. 8D). Furthermore, cycloheximide treatment of A549‐T24 cells with USP24 knocked down reduced the protein stability of the GPX4 and DHFR proteins compared to control (Fig. 8E). Next, we want to investigate whether the degradation of ABC transporters and antioxidants is via autophagy or the ubiquitin‐proteasome system (UPS). We found that chloroquine, which is an autophagy inhibitor, could reverse NCI677397‐induced degradation of ABCG1/5/8, DHFR and GPX4, while MG132, which is a proteasome inhibitor, only reversed that of ABCG5 (Fig. 8F). In summary, NCI677397 leads to protein instability of ABC transporters and antioxidants, implying that ABCG1/5/8, GPX4 and DHFR might be substrates of USP24.

### 3.6 NCI677397 induced therapy‐resistant cancer cell death through ferroptosis

**Fig. 9:**
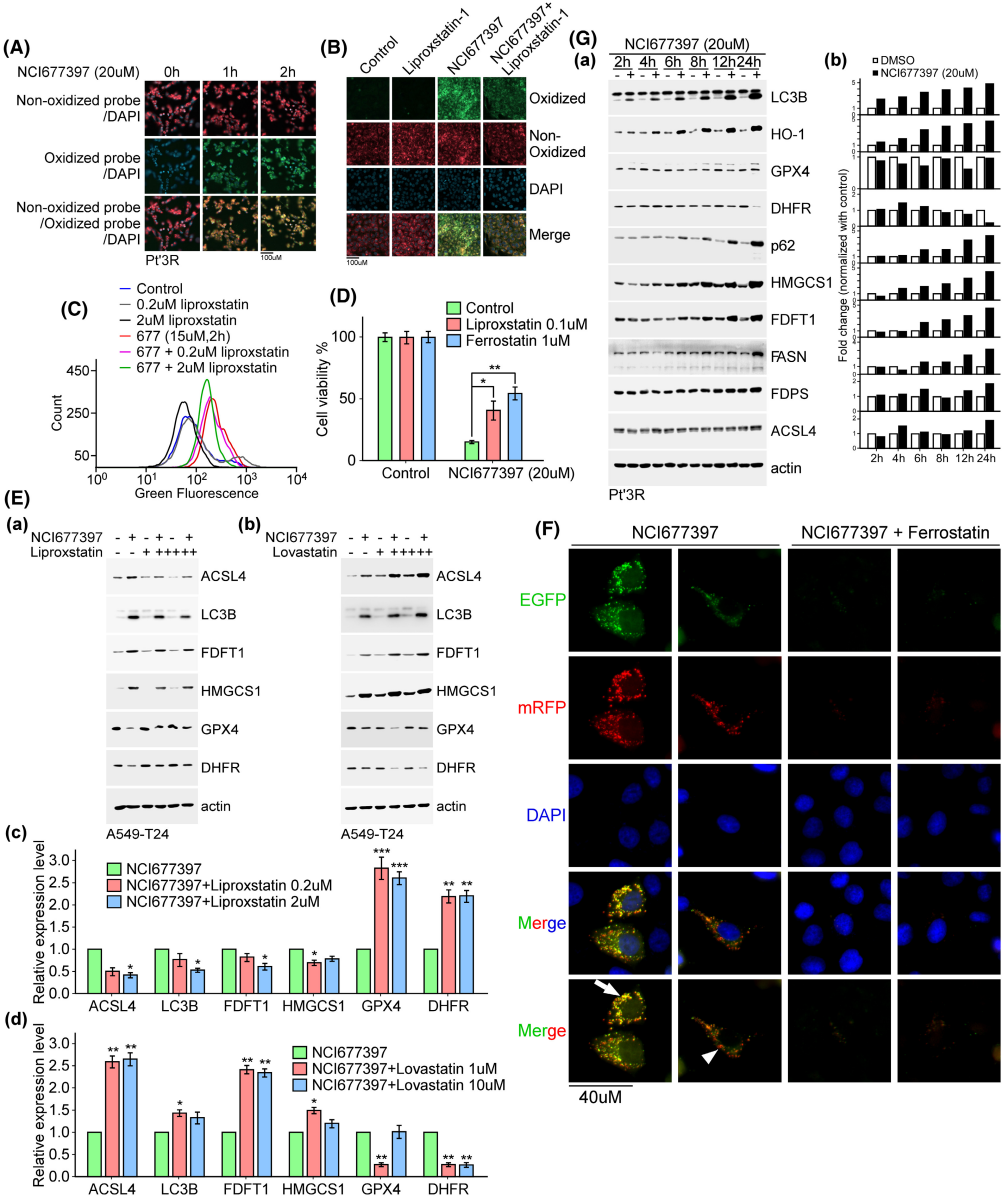
NCI677397‐induced cancer cell death via ferroptosis. (A) After NCI677397 treatment for 1 or 2 h, lipid peroxidation in Pt’3R cells was estimated by staining with C11‐BODIPY^581/591^ reagent. The immunofluorescent signal representing peroxidase lipid (green) was photographed with a fluorescent microscope (*n* = 2). Scale bar, 100 μm. (B, C) After NCI677397, liproxstatin or a combination treatment, cells were stained with C11‐BODIPY^581/591^. Immunofluorescence signals were photographed with a fluorescent microscope (B) or measured by flow cytometry (C) (*n* = 3). Scale bar, 100 μm. (D) Cell viability was studied after NCI677397 treatment or cotreatment with liproxstatin or ferrostatin by CCK‐8 assay. Experiments were performed independently three times, and the data are expressed as the mean ± SEM and statistically analyzed by *t* test: *P < 0.05; **P < 0.01. (E) Cells treated with NCI677397 alone or combination with liproxstatin (a) or lovastatin (b) were collected, and the proteins were measured by western blotting with the indicated antibodies. The indicated proteins (c–d) from three independent experiments were quantitated, the data was expressed as the mean ± SEM and statistically analyzed by *t* test: *P < 0.05, **P < 0.01, ***P < 0.001. (F) mRFP‐EGFP‐LC3 was transfected into A549‐T24 cells and then treated by NCI677397 with or without ferrostatin for studying the signals of mRFP and EGFP via immunofluorescence microscopy. An arrow indicates yellow color and an arrowhead indicates orange color (*n* = 3). Scale bar, 40 μm. (G) Cells treated with NCI677397 for different durations were collected, and western blotting with the indicated antibodies was performed (*n* = 3). The quantitation of the indicated proteins is shown in (b).

We investigated whether NCI677397‐induced cancer cell death occurs via ferroptosis. In this effort, we accidentally discovered that lipid peroxidation occurred immediately after Pt’3R cells were treated with NCI677397, showing an increase in 1 h (Fig. 9A). Thus, we further evaluated the impact of NCI677397 on lipid peroxidation and found that the levels of oxidized lipids were obviously increased in 2 h after NCI677397 treatment and that the levels were reduced when the cells were pretreated with liproxstatin‐1, a ferroptotic inhibitor, as indicated by fluorescence and flow cytometry assays, the results of which are shown in Fig. 9B,C, respectively. To determine whether NCI677397 induces ferroptosis, cell viability was evaluated by CCK‐8 assay. As shown in Fig. 9D, NCI677397 markedly decreased cell viability, but these effects were alleviated by treatment with liproxstatin or ferrostatin, which are ferroptotic inhibitors, demonstrated that NCI677397 induced cell death via ferroptosis. Moreover, we analyzed the molecular mechanism and found that NCI677397 increased the protein levels of ACSL4, LC3B, FDFT1 and HMGCS1 and decreased those of GPX4 and DHFR, but liproxstatin pretreatment reversed the effect of NCI677397, implying that the ferroptotic inhibitor mitigated NCI677397‐mediated cell death (Fig. 9E(a),(c)). Interestingly, we also found that pretreatment with lovastatin, which is a cholesterol biosynthesis inhibitor, amplified the effect of NCI677397, suggesting that blocking cholesterol synthesis exacerbated NCI677397‐mediated cell death (Fig. 9E(b),(d)). Furthermore, we used a pH‐sensitive plasmid, mRFP‐EGFP‐LC3, to study autophagic flux (Fig. 9F). The data indicated that NCI677397‐induced LC3 puncta partially fused with lysosome (orange color, arrowhead), but partially fused without lysosome (yellow color, arrow), which suggested that NCI677397‐induced autophagosome might overload lysosomal capacity. Combined with ferrostatin treatment, a ferroptotic inhibitor, we found that ferrostatin treatment obviously curbed LC3 puncta expression, which hinted that ferroptotic inhibitors might rescue NCI677397‐induced cell death. Next, we investigated the detailed molecular mechanisms induced by NCI677397 treatment using samples collected at different time points. The analysis revealed that LC3B and HO‐1 were instantly upregulated, and GPX4 was downregulated after 2 h of NCI677397 treatment. Moreover, an increase in p62, HMGCS1, FDFT1, FASN and FDPS was found after 6 h of NCI677397 treatment, and finally, ACSL4 was activated and the DHFR level was reduced after 24 h of NCI677397 treatment (Fig. 9G), indicating that after NCI677397 treatment lipid peroxidation/lipid ROS is first induced, followed by the activation of oxidative stress sensors, such as autophagy and HO‐1. At the same time, the GPX4 level is decreased, and then, cholesterol and fatty acid biosynthesis enzyme levels are increased, which promotes the antioxidant maturation needed for cell survival, but causes excessive increases in autophagy, HO‐1 and cholesterol levels, ultimate leading to ferroptosis (Fig. 9G).

## 4 Discussion

**Fig. 10:**
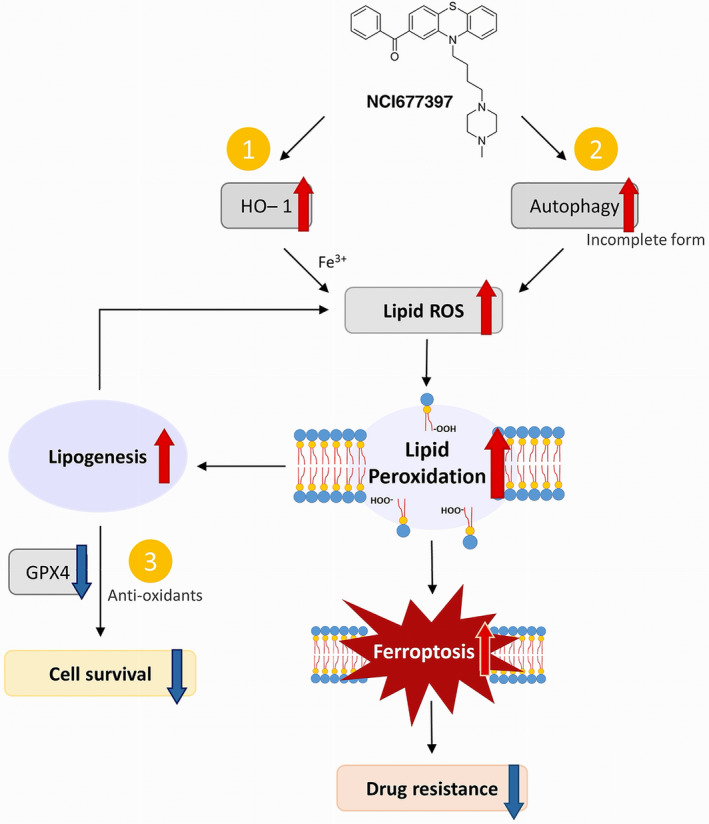
Schematic representation of the working model. In cancer cells, after NCI677397 (a USP24 inhibitor; USP24i) treatment induces lipid ROS production, autophagy is activated and cholesterol biosynthesis induces antioxidant maturation to rescue the cells from ferroptosis. Unfortunately, NCI677397 simultaneously destabilizes antioxidants and ABC transporters, resulting in a cancer cells ferroptosis.

In this study, we discovered that a novel ferroptosis inducer (FIN), NCI677397, which is a USP24‐specific inhibitor, is potentially as a new treatment for drug resistance in cancers. After cancer cells were treated with NCI677397, lipid peroxidation was immediately induced, followed by autophagy activation and HO‐1 upregulation in response to the oxidative stress [46, 47, 48]. Subsequently, cholesterol and fatty acid biosynthesis alleviated ferroptosis by GPX4 maturation or CoQ10 synthesis [45, 49, 50]. Notably, NCI677397 resulted in redox imbalance because it destabilized the GPX4 and DHFR proteins, which might be the substrates of USP24, producing excessive levels of oxidized lipids that drove cancer cells toward ferroptosis (Fig. 10).

Ferroptosis is a form of iron‐catalyzed cell death characterized by a reduction in glutathione peroxidase‐4 (GPX4) activity, which is accompanied by peroxidation of polyunsaturated fatty acids (PUFAs) leading to the accumulation of phospholipid hydroperoxides [51]. Although the relationship between autophagy and ferroptosis is still debated, previous studies have suggested that autophagy is an initiator of ferroptosis mediated through chaperone‐mediated autophagy (CMA) and ferritinophagy mechanisms [52]. However, a recent study demonstrated that phospholipid peroxidation inhibited autophagy by stimulating the ATG4B‐mediated delipidation of peroxidized LC3‐PE, which led to the formation of incomplete autophagosomes [53]. This stimulus by NCI677397 triggered induction of autophagy, but cancer cells cannot be rescued by accelerated autophagy because they form incomplete autophagosomes resulting from the NCI677397‐induced redox imbalance and lipid peroxidation. In this study, we also found that NCI677397 treatment partially seems to induce incomplete autophagosomes (Fig. 9F). Notably, p62 was not degraded via NCI677397‐induced autophagy activation because of the formation of incomplete autophagosomes. On the other hand, HO‐1 metabolizes heme into ferrous iron, carbon monoxide and biliverdin/bilirubin, and it has been suggested that HO‐1 exerts cytoprotective effects under various stress‐related conditions [54]. However, an increasing number of studies have shown a dark deleterious HO‐1 function, in which it acts as a critical mediator in ferroptosis induction and plays a causative role in progression of several diseases [55]. The levels of cellular iron and ROS are the determinative factors of HO‐1 function, with excessive cellular iron and ROS levels tending to divert HO‐1 from playing a protective role and inducing it to play a destructive role [56]. The HO‐1 functions related to cell death may be leveraged to mediate ferroptosis in a chemotherapeutic strategy against tumors [57]. In this study, we found that HO‐1 was immediately upregulated by NCI677397, at the same time that autophagy was activated; therefore, HO‐1 exerted cytoprotective effects against lipid ROS. However, owing to NCI677397‐induced redox imbalance and incomplete autophagosomes, sustained lipid peroxidation led to a constant increase in HO‐1 level, and then, reaching a tipping point, HO‐1 drove cancer cells to undergo ferroptosis. It is noteworthy that recent studies have shown that SLC47A1 acts as an endogenous repressor of ferroptosis, while it is also upregulated by inducers of ferroptosis [38], similar to our results (Fig. 7F). It seems that NCI677397‐induced SLC47A1 upregulation is response to ferroptosis induction; however, the role of SLC47A1 on NCI677397‐induced ferroptosis requires further investigation.

Ferroptosis can be regulated by multiple parallel antioxidant pathways, such as the FSP1/DHODH/CoQ10 axis, GCH1/BH4/DHFR axis and cysteine/GSH/GPX4 axis [58]. The mevalonate pathway is a core metabolic pathway of cholesterol synthesis and is also critical for the maturation of GPX4 and the synthesis of CoQ10 [59, 60]. Cholesterol is an essential lipid component in the mammalian cell membrane, where it maintains membrane fluidity and integrity, and forms membrane microstructures. In this study, NCI677397‐induced cholesterol biosynthesis mediates the maturation of antioxidants that overcome ferroptosis, but cancer cells ultimately undergo ferroptosis programmed cell death. There are two possibilities to explain this phenomenon: NCI677397 causes redox imbalance owing to destabilization of antioxidants, and NCI677397 blocks cholesterol trafficking by decreasing the levels of ABC transporters (ABCG1/5/8). In addition, our recent study showed that USP24 stabilized ABCG2 and P‐gp to promote cancer cell drug resistance [14], implying that USP24 recognizes specific sequences or structures in ABC transporters, especially G family. Moreover, ABC transporters function as pumping toxins or chemotherapy drugs out of cells, leading to drug‐resistant cancer and cholesterol trafficking contributes to the development of drug‐resistant cancer [61, 62], so NCI677397 will be a potential target for treating drug‐resistant cancers.

Increasing evidence shows the potential of triggering ferroptosis for cancer therapy, particularly for eradicating aggressive malignancies that are resistant to traditional therapies [40, 63, 64], suggesting that ferroptosis is a powerful weapon against cancer. Recently, a great deal of effort has been directed to the design and development of anticancer drugs based on ferroptosis induction [65]. Therefore, we think that NCI677397 is a potential therapeutic anticancer drug that induces ferroptotic cell death. In this study, we clarified that USP24 inhibitors induced ferroptosis in different cancer types. On the other hand, a large amount of evidence supports the notion that impaired iron homeostasis and dysregulated metabolic pathways play roles in the progression of liver disease via ferroptosis [66]. Although the molecular mechanisms by which ferroptosis causes disease are poorly understood, several ferroptosis‐associated pathways and genes have been implicated in liver disease. The trans‐differentiation of hepatic stellate cells (HSCs) into matrix‐producing myofibroblasts is considered a key step in the development of liver fibrosis [67]. Recent studies have demonstrated the potential of inducing ferroptosis in HSCs using artemether, artesunate, or magnesium isoglycyrrhizinate as a treatment to mitigate the development of liver fibrosis [68]. Moreover, certain regulators of ferroptosis in HSCs have been reported to be promising targets in preventing liver fibrosis [69]. Taken together, these studies indicate that inducing ferroptosis in HSCs may represent a viable strategy for treating and/or preventing liver fibrosis. In our opinion, the effect of knockdown of USP24 is not as good as USP24 inhibitors, because we could not absolutely restrain the gene expression by knockdown strategy except by using small molecular inhibitors. The reason is that NCI677397 specifically binding to USP24 and blocking its enzyme activity suddenly, while knockdown of USP24 partially decreasing its protein level slowly [14]. Therefore, it is worthy determining whether USP24 inhibitors can be a potential treatment for liver fibrosis as well as drug‐resistant cancers and we will persist in optimizing USP24 inhibitors in the future.

## 5 Conclusions

In conclusion, our finding showed that the specific and novel inhibitor of USP24, NCI677397, induced autophagy and lipid ROS, while NCI677397 simultaneously induced HO‐1 and lipogenesis‐related genes, such as FASN, HMGCS1, FDFT1 and FDPS, and reduced ABC transporters (ABCG1/5/8) and antioxidants, such as GPX4 and DHFR. Ultimately, NCI677397 gave rise to lipid peroxidation and ferroptotic cell death in drug‐resistant cancer cells.

## Conflict of interest

The authors declare no conflict of interest.

## Author contributions

S‐AW, F‐MY, KZ and J‐JH designed the experiments and interpreted the result, S‐AW, Y‐CW and F‐LH conducted the experiments, S‐AW, KZ and J‐JH wrote the manuscript.

### Peer review

The peer review history for this article is available at https://www.webofscience.com/api/gateway/wos/peer‐review/10.1002/1878‐0261.13574.

## Supporting information


**Table S1.** Sequences of primers used for Real time‐PCR.

## Data Availability

RNA‐Seq, MS proteomics and lipidomics data were shown in Figs 4, 5 and 7. All data generated or analyzed during the current study are available from the corresponding author upon reasonable request.
