# Supplementary material for: NCI677397 targeting USP24‐mediated induction of lipid peroxidation induces ferroptosis in drug‐resistant cancer cells
Source: Mol Oncol. 2023 Dec 30;18(9):2255–76. doi: 10.1002/1878-0261.13574 (PMC11467797; doi:10.1002/1878-0261.13574)
Supplement: Supplementary file 1 — Table S1. Sequences of primers used for Real time‐PCR. [file MOL2-18-2255-s001.docx]

Supplementary Table 1

Sequences of primers used for Real time-PCR

| Gene | Forward/Reverse | Sequence 5' to 3' |
| --- | --- | --- |
| HMGCS1 | F | CAAAAAGATCCATGCCCAGT |
|  | R | AAAGGCTTCCAGGCCACTAT |
| FDFT1 | F | ATAACCAATGCACTGCACCA |
|  | R | CCTTTCCGAATCTTCACTGC |
| DHCR7 | F | CATTGACATCTGCCATGACC |
|  | R | ACAGGTCCTTCTGGTGGTTG |
| LSS | F | TCCCGGACTATCTCTGGATG |
|  | R | ACCTGTGAGAGCCTCAGGAA |
| IDI1 | F | CCGAGCTTGAGGAAAGTGAC |
|  | R | CATGTTCACCCCAGATACCA |
| SQLE | F | GTCTCCGGAAAGCAGCTATG |
|  | R | AAAAGCCCATCTGCAACAAC |
| ACAT2 | F | CCGGAAGATGTGTCTGAGGT |
|  | R | CACAGCTTTTAGGCCTGACC |
| SCD | F | CCCAGCTGTCAAAGAGAAGG |
|  | R | CAAGAAAGTGGCAACGAACA |
| MVD | F | AGGACAGCAACCAGTTCCAC |
|  | R | GTGTCGTCCAGGGTGAAGAT |
| FASN | F | AGTACACACCCAAGGCCAAG |
|  | R | GTGGATGATGCTGATGATGG |
| HMGCR | F | GTCATTCCAGCCAAGGTTGT |
|  | R | TCCTGTCCACAGGCAATGTA |
| ACLY | F | ATCTCCGGCCTCTTCAATTT |
|  | R | ACTCGATGTCACCCCACTTC |
| DHCR24 | F | TGTTGCCTGAGCTTGATGAC |
|  | R | GACCAGGGTACGGCATAGAA |
| LDLR | F | GTGCTCCTCGTCTTCCTTTG |
|  | R | TAGCTGTAGCCGTCCTGGTT |
| STARD4 | F | CTGCTGGTCAGCTTTGGAAT |
|  | R | ACCAACCACAGGGATGGTTA |
| SC5D | F | TGTCCCCCAAATCTTACAGC |
|  | R | CGCTTTCCCTCTGTCATCTC |
| MVK | F | GCTCAAGTTCCCAGAGATCG |
|  | R | ATGGTGCTGGTTCATGTCAA |
| ACACA | F | ACCACCAATGCCAAAGTAGC |
|  | R | CTGCAGGTTCTCAATGCAAA |
| FADS2 | F | ACCTGCCCTACAATCACCAG |
|  | R | AGGTGATGAAGAACCGGATG |
| FADS1 | F | ACTGGTTTGTGTGGGTGACA |
|  | R | GGGAAAAAGATGGTGCTCAA |
| ACSS2 | F | GAACATGAGGCTGTTGCAGA |
|  | R | ATGGGGCCAATCTTTTCTCT |
| SREBF2 | F | TGGCTTCTCTCCCTACTCCA |
|  | R | GAGAGGCACAGGAAGGTGAG |
| INSIG1 | F | GGTGGACATTTGATCGTTCC |
|  | R | TGACGCCTCCTGAGAAAAAT |
| NPC1 | F | GGTCCGCCTGTGTACTTTGT |
|  | R | GGCTTCACCCAGTCGAAATA |
| NPC2 | F | TTCCTGGCAGCTACATTCCT |
|  | R | TGCTGGTGAAGGTGACATTG |
| LRP1 | F | AGCAAACGAGGCCTAAGTCA |
|  | R | GCTGCTTGTGCTGATGGTAA |
| ACSL3 | F | TGTTTCTGCTGTCCTGTTGG |
|  | R | CTGGGGTGTGGCTTATCAGT |
| ACSL4 | F | AATGCAGCCAAATGGAAAAG |
|  | R | CACAGAAGATGGCAATGGTG |
| SQSTM1/ p62 | F | CACCTGTCTGAGGGCTTCTC |
|  | R | CGATGGACCAGAAGCTGATT |
| HMOX1/ HO-1 | F | ATGACACCAAGGACCAGAGC |
|  | R | GTGTAAGGACCCATCGGAGA |
